# Canine transposition as an alternative to trauma of the maxillary incisors: case report

**DOI:** 10.1590/2177-6709.23.4.055-063.oar

**Published:** 2018

**Authors:** María Salcedo Lara, Rocío Briones Rodríguez, María Biedma Perea, Beatriz Solano Mendoza

**Affiliations:** 1 Universidad de Sevilla, Facultad de Odontología, Departamento de Ortodoncia (Sevilla, España). Universidad de Sevilla Universidad de Sevilla Facultad de Odontología Departamento de Ortodoncia Sevilla Spain; 2 Universidad de Sevilla, Facultad de Odontología, Departamento de Pediatría (Sevilla, España). Universidad de Sevilla Universidad de Sevilla Facultad de Odontología Departamento de Pediatría Sevilla Spain

**Keywords:** Dental transposition, Orthodontics

## Abstract

**Objective:**

The present article aims at reporting the clinical case of a patient who suffered trauma at two years of age, causing almost complete apical displacement of the deciduous maxillary left central incisor and of the permanent incisor.

**Methods:**

Ectopia secondary to intrusion was minimized by surgical removal of the ectopic tooth, and the left permanent canine was submitted to orthodontic traction to replace the extracted tooth.

**Results:**

The treatment period lasted 36 months, resulting in correct occlusion and a good aesthetic outcome.

**Conclusions:**

Dental transposition carried out by means of orthopedic traction is a good alternative in cases of a very unfavorable ectopic tooth position.

## RESUMO

**Objetivo:**

o presente relato de caso descreve uma paciente que sofreu trauma aos dois anos de idade, causando deslocamento apical quase completo do incisivo central esquerdo decíduo e do incisivo permanente.

**Métodos:**

a ectopia resultante da intrusão foi minimizada pela remoção cirúrgica do dente ectópico, e o canino permanente esquerdo foi tracionado ortodonticamente para substituir o dente extraído.

**Resultados:**

o período de tratamento durou 36 meses, obtendo-se uma oclusão correta e um bom resultado estético.

**Conclusões:**

a transposição dentária realizada através da tração ortopédica é uma boa alternativa em casos de dentes ectópicos em posições muito desfavoráveis.

## INTRODUCTION

In many cases, tooth eruption does not follow a correct chronological sequence, due to trauma causing alterations in the natural eruption sequence of the permanent teeth^1^. Intrusion luxation of the deciduous tooth, i.e., displacement of the tooth within the alveolar bone, occurs when impaction takes place in the apical direction along the longitudinal axis of the tooth. The most frequent result of such trauma is the buccal displacement of the apex, away from the space reserved for the permanent tooth germ.^1^ However, when intrusion occurs in the opposite direction, i.e., when the root inclines to lingual, there is a clear risk of damage to the permanent tooth germ. One of the effects of deciduous tooth intrusion upon the permanent tooth germ is ectopic eruption. This is due to physical displacement of the germ at the time of trauma, giving rise to serious eruption disorders.^2^^,^^3^

Transposition can be described as the positional exchange of two adjacent or non-adjacent teeth, especially in relation to their roots - the estimated prevalence is 0.3%. The most commonly affected teeth are maxillary canines, with a palatine position.^4^^,^^5^

The management of such cases, which are frequently associated to different eruption anomalies, requires a multidisciplinary approach (surgical, orthodontic and aesthetic) in order to restore function and aesthetics in growing patients. 

Thus, the present case report describes a patient with multiple eruption disorders due to a deciduous tooth intrusion at two years of age. This resulted in delayed eruption and ectopia of the maxillary left central incisor, accompanied by anomalous eruption of the adjacent canine, which was successfully resolved by a multidisciplinary management approach.

## DIAGNOSIS

A 9-year-old girl in the second mixed dentition phase sought orthodontic treatment at the Department of Orthodontics of University of Seville (Spain) due to delayed eruption of the maxillary left central incisor. All necessary radiographs (panoramic and lateral cephalometric radiographs) for establishing the diagnosis were obtained. The patient had no family history of interest, and had suffered trauma at two years of age, causing intrusion of the deciduous maxillary left central incisor. 

**Figure 1:**
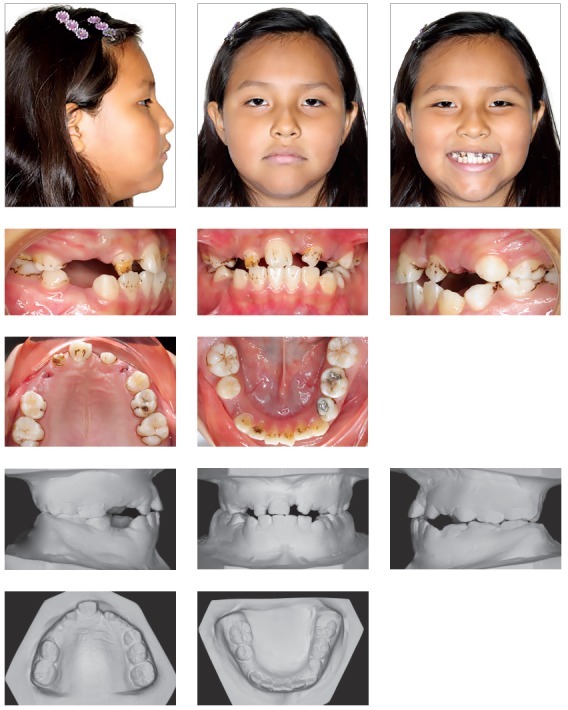
Pre-treatment facial and intraoral photographs, and pretreatment dental casts.

The clinical examination revealed a meso-dolichofacial facial pattern, with facial asymmetry, due to a diminished facial lower third and a flattened nose (Fig 1). The profile was convex (159^o^), with a noticeable nasolabial angle (94^o^) and a short cranial base (58.4 mm). 

In the intraoral examination, the maxillary left central incisor was absent, with bilateral Class I molar relationship, small overjet, edge-to-edge overbite and a 3-mm midline deviation to the left side^6^ (Fig 1).

**Figure 2:**
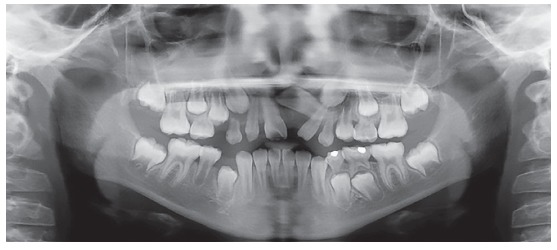
Pre-treatment panoramic radiograph.

The panoramic radiograph study confirmed the anomalous position of the maxillary left central incisor, secondary to intrusive luxation of the deciduous predecessor (Fig 2). The ectopic incisor was in a crosswise position, with its crown at the level of the right central incisor apex, and its apex at the level of left canine, besides left lateral incisor crown moved towards the central incisor space. In addition, the maxillary left canine did not show a correct natural eruption sequence, being positioned at the level of lateral incisor apex, and not at deciduous canine level. Third molar germs were not observed, and the maxillary and mandibular premolars eruption was complete.

**Figure 3:**
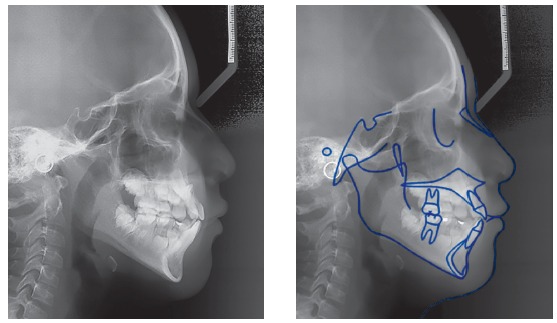
Pre-treatment lateral cephalometric radiograph and tracing.

With regard to the cephalometric parameters, the patient showed a slight skeletal Class II pattern of maxillary origin (SNA = 87^o^, SNB = 81^o^, ANB = 6^o^), increased mandibular plane angle (SN.GoGn = 39^o^), and protrusion of both maxillary and mandibular incisors (Ui/a-Pg = 8, Li/ A-Pg = 5.6) (Fig 3).

## TREATMENT OBJECTIVES

Treatment focused on removing the non-viable ectopic teeth and facilitating viable ectopic eruption of the remaining teeth, even if not adopting their correct position in the dental arch - thereby ensuring adequate aesthetics and function.

## TREATMENT OPTIONS

The first therapeutic option was maxillary left central incisor auto-transplantation, consisting of surgically positioning the tooth in its alveolar cavity.^7^ Auto-transplantation would have offered the advantage of preserving the central incisor, though this option was rejected, due to the absence of alveolar bone available for transplantation into this area.^4^

Another option was extraction of the ectopic maxillary central incisor and placement of a Maryland bridge^8^^,^^9^ until the completion of growth. This alternative would allow preservation of the other teeth in the correct position, though the premature age of the patient and bone plate loss would impede aesthetic implantation which, in turn, could pose risks for lateral left incisor revitalization due to incorrect left canine angulation.

As a third option after ectopic central incisor extraction, transposition of the maxillary left canine^10^ into the left central incisor position by means of orthodontic traction was considered, followed by premolar transformation into canine, and canine into central incisor. This strategy would involve premolar extraction in the first, third and fourth quadrants, making the case symmetrical and stabilizing the midline with respect to the facial line. This was the selected orthodontic treatment plan, since it allowed correct occlusion, by closing the anterior open bite, and thus afforded adequate aesthetic results without hindering left lateral incisor revitalization.

## TREATMENT PROGRESS

**Figure 4:**
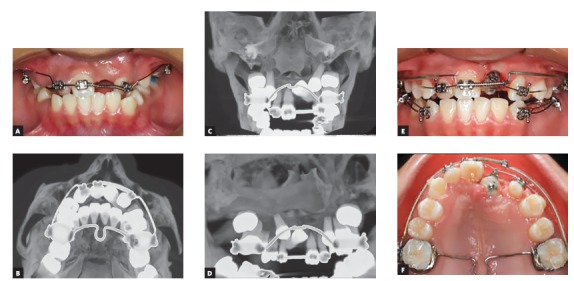
A, B) Space opening and CAT scan with inclusions; C, D) traction of the canine into the position of left central incisor and E, F) extractions of right and left mandibular first premolars.

To prepare the maxillary left canine space, a sectional 0.014-in NiTi wire was placed and subsequently an 0.016 x 0.022-in blue Elgiloy^®^ utility archwire with opened coil spring between the right maxillary central incisor and the left maxillary lateral incisor. At the same time, a transpalatal bar was positioned to increase posterior anchorage during canine traction (Fig 4). 

Surgery was performed for left canine traction with an open surgical technique, and a button was placed in its palatine surface. Once the tooth had partially erupted, the maxillary central incisor bracket was positioned on the maxillary left canine buccal surface (Fig 4). Once the canine was moved to the central incisor position, the contralateral maxillary first premolar and both lower first premolars were extracted, with distalization of the left canine, for midline centralization and canine Class I occlusion.

**Figure 5:**
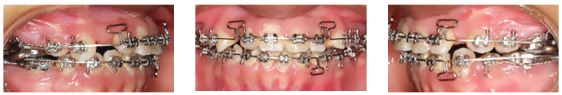
Anterior retrusion.

Space closure was carried out by means of a T-arch with transpalatal bar in the maxillary dental arch and DKL (double keyholed looped) in the mandibular dental arch (Fig 5). Continuing the treatment, maxillary anterior sector restoration phase was carried on, using composite by means of a stratified technique,^11^^,^^12^ with micro-aesthetic photographs and waxing diagnosis used to examine dental proportion, gingival heights and color.^13^^,^^14^ Based on diagnostic waxing, it was observed the need to increase the mesiodistal size of the right and left maxillary lateral incisors, modify the anatomy of the left maxillary canine, which was transformed into central incisor, and of the left maxillary first premolar, which was transformed into canine. Fixed retention was applied from the right maxillary second premolar to the left maxillary second premolar, and from the right mandibular canine to the left mandibular canine. Upper and lower removable retentions were placed, by means of a wraparound retainer. The wraparound retainer facilitates a greater number of occlusal contacts during retention, by vertical movements of the pieces of posterior sectors. This type of retention protocol was placed to avoid undesirable relapse movements in the short-term, until any other more effective method to prevent relapse would be available^15^. 

## TREATMENT OUTCOME

**Figure 6:**
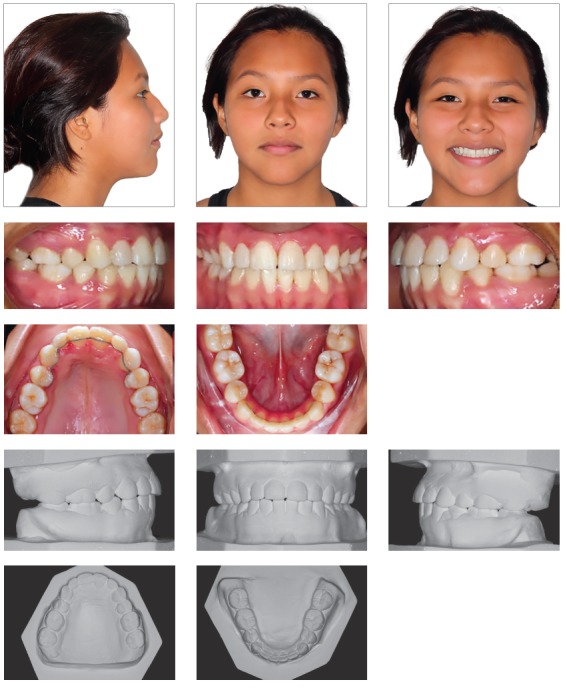
Post-treatment facial and intraoral photographs and dental casts.

The post-treatment photographs showed a profile with good projection of the chin and correct lip sealing despite an increase in the lower facial third. With regard to occlusion, it was achieved a canine and molar Class I occlusion, with correct overjet and overbite, taking into account that the patient had an edge-to-edge relationship (Fig 6).

**Figure 7:**
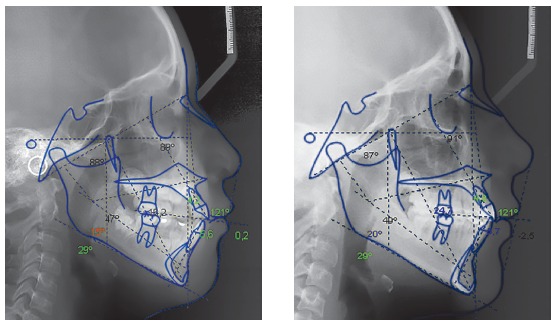
Pre and post-treatment cephalometric tracings.

**Figure 8:**
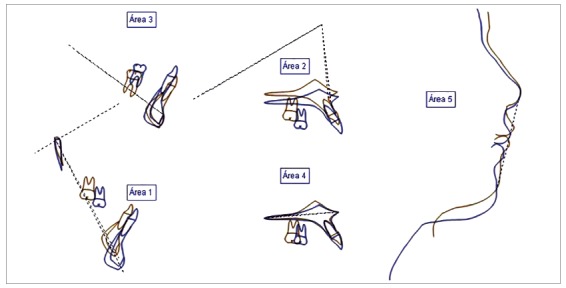
Superimposition of the initial and near end of treatment cephalometric tracings.

**Figure 9:**
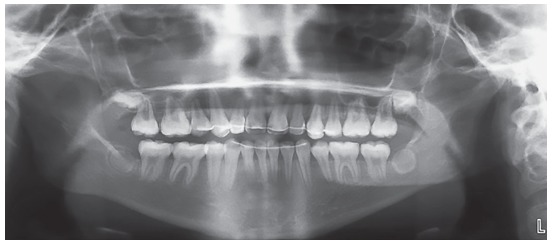
Post-treatment panoramic radiograph.

Acceptable dental aesthetics was achieved using a stratified technique with composite and gingival recontouring. Superimposition of the radiographs showed favorable growth with minimal changes in incisor inclination, due to the treatment with extractions (Figs 7 and 8). The panoramic radiographs showed root parallelism with slight root angulation of the right maxillary canine, typical of the anatomy of this tooth. Moreover, diminished root length was observed due to the premature start of orthodontic treatment without apex closure (Fig 9).

## RETENTION RECORDS

**Figure 10:**
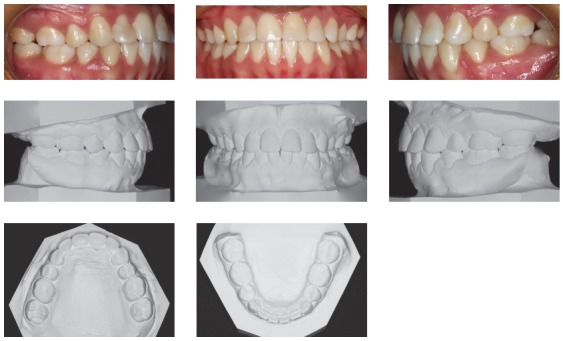
One year retention intraoral photographs and dental casts.

One year after treatment, the patient showed no occlusive functional changes, maintaining molar and canine Class I occlusion and correct overjet and overbite (Fig 10).

## DISCUSSION

In many cases, when deciduous teeth suffer severe luxation, the permanent teeth can experience genuine eruption problems, including even arrested or aborted eruption.^16^^,^^17^

In intrusive trauma, the permanent tooth often remains lodged in the maxillary external plate as a consequence of the trauma. This prevents adequate deciduous tooth root resorption, required for correct dental replacement.^18^

One infrequent indication of auto-transplantation is dental ectopia or retention,^19^ usually in cases where is not possible to perform orthodontic traction due to the position of the tooth. However, this treatment option was not viable, since in order for auto-transplantation to be successful, certain preliminary conditions are required,^20^ including active root development, which had already been concluded in this patient.

Moorres et al^21^ proposed a classification of the root development stages, and together with Andreasen et al,^22^ demonstrated that the greatest success rates are obtained when auto-transplantation is performed in the third and fourth stages (one-half and three-quarters of root formation, respectively). Therefore, with respect to the present clinical case, success would have been unlikely, since the central incisor showed complete root formation, which would require consistent pulp treatment.^23^ Another determining condition for auto-transplantation is the existence of enough alveolar bone in the receptor area to accommodate the donor germ. Such bone was practically non-existent in this case.^24^^,^^25^

In view of the above, it was decided to accept canine transposition^4^^,^^10^ in a central position, since the direction of eruption was favorable. The anatomy of the canines offers a relative possibility for simulating a central incisor, since the mesiodistal width is more similar than that of a lateral incisor, in the same way as the vertical height and gingival margin.

Another treatment option was to keep the central space with a Maryland bridge,^8^^,^^9^ followed by implant positioning, since it does not require premolar extractions and is a minimally invasive technique, affording good aesthetic outcomes and acceptable predictability over the middle term^24^. However, this option was dismissed because the patient exhibited dental biprotrusion and diminished overbite, as well as incorrect left maxillary canine eruption with possible involvement of the apex of the left maxillary lateral incisor. Furthermore, it must be kept in mind that the early loss of a tooth results in bone loss; consequently, at the time of implant placement, *en bloc* bone grafting would be required.^26^

## CONCLUSIONS

The results obtained highlight the importance of a multidisciplinary approach in order to establish a correct diagnosis and afford optimum treatment for patients like this. On the other hand, dental transposition carried out by means of orthodontic force is a good alternative in cases of very unfavorable ectopic tooth position. 
